# Synthesis, Cytotoxicity and Mechanistic Evaluation of 4-Oxoquinoline-3-carboxamide Derivatives: Finding New Potential Anticancer Drugs

**DOI:** 10.3390/molecules19056651

**Published:** 2014-05-22

**Authors:** Luana da S. M. Forezi, Nathalia M. C. Tolentino, Alessandra M. T. de Souza, Helena C. Castro, Raquel C. Montenegro, Rafael F. Dantas, Maria E. I. M. Oliveira, Floriano P. Silva, Leilane H. Barreto, Rommel M. R. Burbano, Bárbara Abrahim-Vieira, Riethe de Oliveira, Vitor F. Ferreira, Anna C. Cunha, Fernanda da C. S. Boechat, Maria Cecília B. V. de Souza

**Affiliations:** 1 Outeiro de São João Batista, Fluminense FederalUniversity (UFF), s/n, Niterói 24020141, RJ, Brazil; 2 Laboratory of Molecular Modeling & QSAR (ModMolQSAR), Federal University of Rio de Janeiro (UFRJ), Rio de Janeiro 21949-900, RJ, Brazil; 3 LABIEMol, Outeiro de São João Batista, Fluminense Federal University, s/n, Niterói 24020-141, RJ, Brazil; 4 Institute of Biological Sciences, Federal University of Pará, Av. Augusto Corrêa, n.01—Guamá, Belém, 66075-110, Pará, Brazil; 5 Laboratory of Biochemistry of Proteins and Peptides, Oswaldo Cruz Institute, FIOCRUZ, Rio de Janeiro 21040-900, RJ, Brazil

**Keywords:** 4-oxoquinoline, carboxamide, heterocycles, anticancer

## Abstract

As part of a continuing search for new potential anticancer candidates, we describe the synthesis, cytotoxicity and mechanistic evaluation of a series of 4-oxoquinoline-3-carboxamide derivatives as novel anticancer agents. The inhibitory activity of compounds **10**–**18** was determined against three cancer cell lines using the MTT colorimetric assay. The screening revealed that derivatives **16b** and **17b** exhibited significant cytotoxic activity against the gastric cancer cell line but was not active against a normal cell line, in contrast to doxorubicin, a standard chemotherapeutic drug in clinical use. Interestingly, no hemolytical activity was observed when the toxicity of **16b** and **17b** was tested against blood cells. The *in silico* and *in vitro* mechanistic evaluation indicated the potential of **16b** as a lead for the development of novel anticancer agents against gastric cancer cells.

## 1. Introduction

Currently, most treatments against cancer are multimodal, involving chemotherapy, radiation and surgery to treat tumors. However, due to the present limitations associated with standard chemotherapy, including side effects and acquired tumor resistance, there is an urgent need to discover new anticancer agents with improved therapeutic profiles. Despite these issues, chemotherapy continues to be the most prevalent pharmacological approach for the treatment of cancer [1].

Oxoquinolines are a class of compounds with important biological activities [2,3,4]. They represent an important group of heterocyclic compounds because of their pharmacological activities against bacterial infections [5].

The mechanism of the antibacterial activity of 4-oxoquinolines involves modulation of prokaryotic type II topoisomerases (DNA gyrase and topoisomerase IV), and they cause cell death by generating high levels of double-stranded DNA breaks. These enzymes are homologous to human type II topoisomerases, which modulate the topological state of the genetic material by passing an intact DNA helix through a transient double stranded break generated in a separate part of DNA. Thus, 4-oxoquinolines may also exhibit anticancer activity through the same mechanism [6,7,8,9].

In the last several decades, the described new 4-oxoquinoline derivatives were able to reduce mortality when administered as prophylaxis for infections in cancer patients [6,7,10,11,12,13] and with feasible anticancer profile. According to the literature [10], the mechanism of action may be related to the inhibition of mammalian topoisomerase II, which is a target of many antitumor agents. Interestingly, some 4-oxoquinolines show antineoplastic activity as high as etoposide, an anticancer drug.

**Figure 1:**
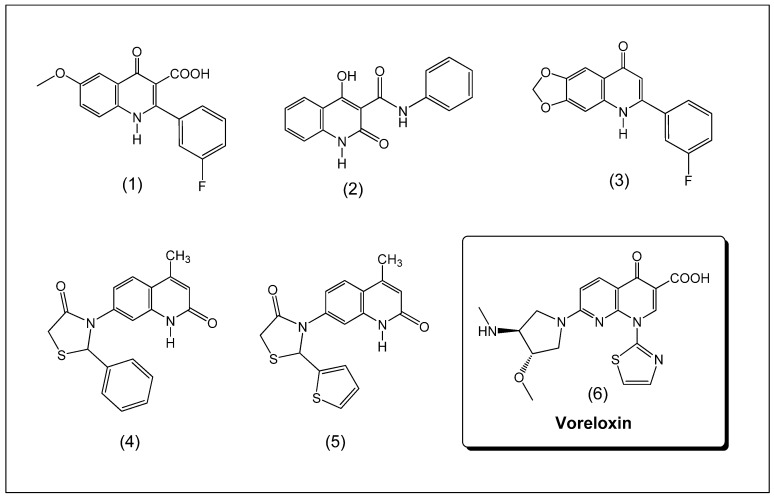
Structures of voreloxin (**6**) and several oxoquinoline derivatives **1**–**5** with anticancer activity.

Some oxoquinoline derivatives and analogues have shown interesting antimitotic profiles (Figure 1) [14,15]. Notably, voreloxin (**6**) [8,16] is an 4-oxoquinoline analogue that shows anticancer activity by intercalating in DNA and affecting topoisomerase II [6,7,10]. Currently, this compound is undergoing pre-clinical evaluation [8,16].

In the continuing search for more selective anticancer agents, many research groups worldwide are conducting research concerning structural modification of the oxoquinolinic core to obtain more potent drugs with fewer side effects. Herein, we report the synthesis, the biological and theoretical evaluations of a series of 4-oxoquinoline derivatives as investigational anticancer agents and explore the mechanism of action of these molecules.

## 2. Results and Discussion

### 2.1. Chemistry

**Scheme 1:**
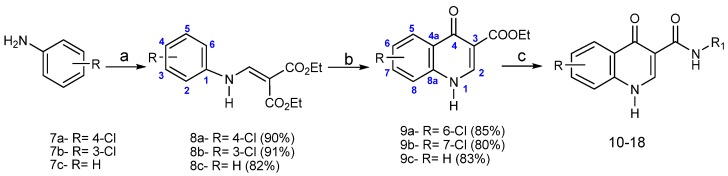
Synthesis of 4-oxoquinolines **10**–**18**.

As shown in the Scheme 1, the derivatives were synthesized using a three-step procedure that involves the condensation of anilines **7** with diethyl ethoxymethylenemalonate (EMME)in refluxing ethanol followed by thermal cyclization of the aniline acrylate intermediates **8**, according to Gould-Jacobs methodology [17,18,19,20,21]. A nucleophilic substitution reaction between oxoquinolines **9** and the appropriate amines in diphenyl ether as solvent affords the respective carboxamides **10**–**18** (Table 1) in 30%–98% yields. Their structures (new compounds) were confirmed by IR, NMR and mass spectroscopy. The HPLC analysis of **16b** and **17b** was also performed.

*Reagents and conditions*: (**a**) diethyl ethoxymethylenemalonate (EMME), ethanol, reflux; (**b**) diphenyl ether, reflux; (**c**) diphenyl ether, amine, 210 °C.

### 2.2. Evaluation of Anticancer Activity in Vitro

All oxoquinolinecarboxamides **10**–**18** were evaluated *in vitro* against three cancer cell lines from different origins: colon (HCT-116), stomach (ACP03) and breast (MDAMB-231). The toxicological profiles of the active derivatives (IC_50_ < 20 μM) were also evaluated by testing on a normal fibroblast cell line (MRC-5) and erythrocytes. The concentration that inhibits 50% of cell growth (IC_50_) was reported in μM, and the hemolytic potential is expressed in μg/mL (Table 2). Finally, a total of 24 derivatives were evaluated, and doxorubicin [22] was used as a positive control.

**Table 1:** Yields and melting points of 4-oxoquinolines **10**–**18**.

Derivative	R	R_1_	Yield (%)	MP (°C)
**10a**	H	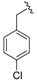	96	244–246
**10b**	6-Cl		85	262–263
**10c**	7-Cl		79	220–221
**11a**	H	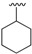	30	209–212
**11b**	6-Cl		58	140–142
**11c**	7-Cl		75	140–141
**12a**	H	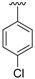	96	>300
**12b**	6-Cl		98	>300
**13a**	H	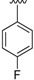	94	>300
**13b**	7-Cl		80	>300
**14a**	H	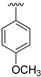	86	>300
**14b**	7-Cl		87	270–273
**15a**	H	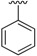	79	>300
**15b**	6-Cl		85	>300
**15c**	7-Cl		83	>300
**16a**	H	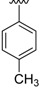	94	>300
**16b**	6-Cl		96	>300
**16c**	7-Cl		86	>300
**17a**	H	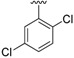	66	>300
**17b**	6-Cl		98	>300
**17c**	7-Cl		63	>300
**18a**	H	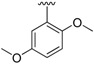	57	250–251
**18b**	6-Cl		44	256–258
**18c**	7-Cl		45	261–262

**Figure 2:**
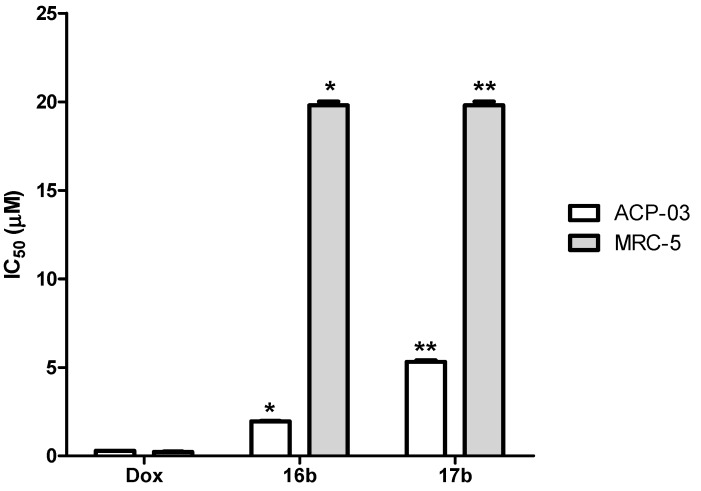
Comparison of the IC_50_values of derivatives against a gastric cancer cell line (ACP-03) and normal fibroblast cell line (MRC-5).

Derivatives **16b** and **17b** displayed cytotoxicity against the gastric cancer cell line, with IC_50_ values of 1.92 and 5.18 μM, respectively (Table 2). However, in normal fibroblasts, they did not display cytotoxicity at 20 µM. Although the IC_50_ of doxorubicin was lower than those of **16b** and **17b**, these derivatives were ten times more selective against cancer cells than to normal cells (Figure 2); doxorubicin shows no selectivity between cancer and normal cells (Table 2 and Figure 2).

Hemolytic activity is an acute toxic effect that must be analyzed when evaluating any new oral or intravenous drug. Therefore, we performed a hemolytic assay in mice erythrocytes to evaluate nonspecific damage to plasma membranes. Importantly, no hemolytic activity (EC_50_ > 200 µg/mL) was observed for any of the tested derivatives, suggesting that the cytotoxicity against cancer cell lines is not related to membrane damage.

**Table 2:** Cytotoxic activity of **10**–**18** on cancer cell lines (ACP-03, HCT-116 and MDA-MB-231), a normal fibroblast cell line (MRC-5) and erythrocytes (to measure hemolysis).

Derivatives			IC_50_ µM		Hemolysis (μg/mL)
	ACP-03	HCT-116	MDAMB231	MRC5	
**10a**	>10	>10	>10	ND	>200
**10b**	>10	>10	>10	ND	>200
**10c**	>10	>10	>10	ND	>200
**11a**	>10	>10	>10	ND	>200
**11b**	>10	>10	>10	ND	>200
**11c**	>10	>10	>10	ND	>200
**12a**	>10	>10	>10	ND	>200
**12b**	>10	>10	>10	ND	>200
**13a**	>10	>10	>10	ND	>200
**13b**	>10	>10	>10	ND	>200
**14a**	>10	>10	>10	ND	>200
**14b**	>10	>10	>10	ND	>200
**15a**	>10	>10	>10	ND	>200
**15b**	>10	>10	>10	ND	>200
**15c**	>10	>10	>10	ND	>200
**16a**	>10	>10	>10	ND	>200
**16b**	**1.92 (1.39–2.66)**	**>10**	**>10**	**>20**	**>200**
**16c**	>10	>10	>10	ND	>200
**17a**	>10	>10	>10	ND	>200
**17b**	**5.18 (3.61–7.45)**	**>10**	**>10**	**>20**	**>200**
**17c**	>10	>10	>10	ND	>200
**18a**	>10	>10	>10	ND	>200
**18b**	>10	>10	>10	ND	>200
**18c**	>10	>10	>10	ND	>200
DOXORUBICIN	0.274 (0.22–0.33)	0.1 (0.047–0.28)	0.43 (0.36–0.52)	0.2 (0.16–0.25)	>200

Data are presented as the IC_50_ values, and 95% confidence intervals were obtained by nonlinear regression for all cell lines (gastric (ACP03), colon (HCT-116), breast (MDAMB231), and normal human fetal lung fibroblast (MRC5)) from three independent experiments. Doxorubicin (Dox) was used as the positive control. Experiments were performed in triplicate. IC_50_ = concentrations that result in 50% inhibition of cell growth, in μM. ND—Not determined.

### 2.3. In Silico Mechanism Analysis: Topoisomerase II as a Feasible Target

**Figure 3:**
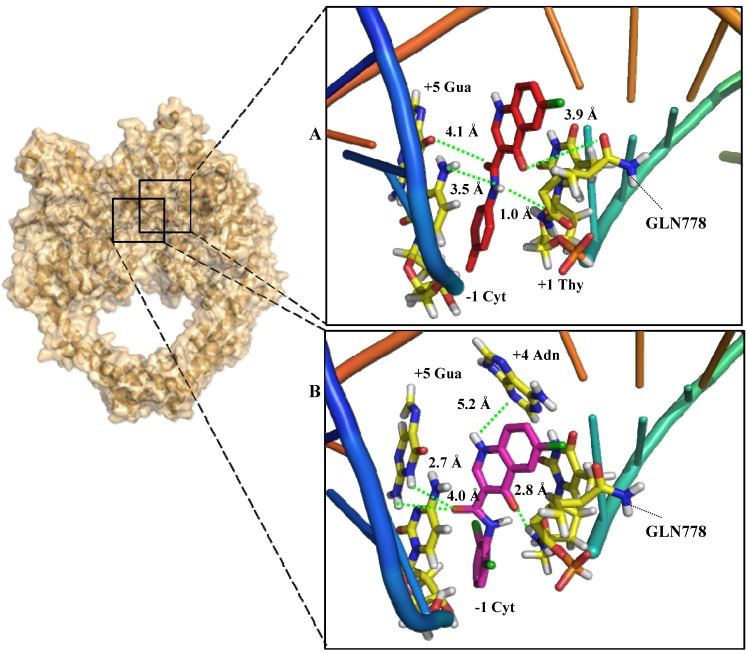
Molecular docking of **16b** (**A**) and **17b** (**B**) in the topoisomerase II binding site: The cartoon-and-stick representation shows the insertion of **16b** (in red) and **17b** (in pink) as well as the hydrogen bonds (in green) between the GLN778 residue of the enzyme (in yellow) and the nucleotides(Adn, adenosine; Cyt, cytosine; Thy, thymine; Gua, guanine).

Previous studies have shown that voreloxin [8,16], a first-in-class cytotoxic 4-oxoquinoline analogue, intercalates into DNA and inhibits topoisomerase II [6,7]. Based on these results, we investigated the feasibility of this mechanism by docking the most actives derivatives **16b** and **17b**, into the DNA binding site of topoisomerase II (Figure 3).

* *p* < 0.001, ** *p* < 0.05, ANOVA (two way), followed by Bonferroni test.

The validity of the docking accuracy was evaluated by redocking using the crystal structure of topoisomerase II (PDB ID: 3QX3 complexed with etoposide, an inhibitor) as described in the experimental section. The reliability of the docking protocol was first checked by comparing the best docking position of the inhibitor with its crystal structure that was obtained using the GOLD program.

The comparison of the redocking results with the co-crystallized conformation was performed using the program Pymol. The *in silico* analysis revealed a conformation similar to the crystallized structure with a root mean square deviation (RMSD) of 0.14 Å. These data supported the hypothesis that the experimental binding mode could be accurately reproduced using this protocol.

The molecular docking data showed that the carbonyl group in the heterocyclic ring of **16b** interacts via a hydrogen bond with the GLN778 residue of the enzyme (O-O 3.9 Å) (Figure 3A) and the same ring of **17b** interacts (O-N 2.8 Å). Similarly, hydrogen bond interactions were also observed between the inhibitor and cytosine (−1), guanine (+5) and thymine (+1) at distances of 3.5, 4.1 and 5.3 Å, respectively for **16b**, while for **17b** were 2.7, 4.0 and 5.2Å between cytosine (−1), guanine (+5), adenosine (+4), respectively. The literature reports that etoposide interacts with the enzyme and with DNA [23]. Interestingly, whereas etoposide interacts through extensive contacts, **16b** and **17b** have a smaller molecular volume and interacts only with the GLN778 residue of the enzyme. This interaction seems to contribute to the stabilization of the complex formed by this ligand and the topoisomerase II. Similar to etoposide [24], these derivatives bind between the base pairs showed, possibly preventing their stacking and consequently blocking the re-ligation of the cleaved phosphodiester bond between the nucleotides. However, **17b** showed no parallel position interactions when compared with **16b**, probable due to the effect of ortho-chlorine substitution. Thereat, weak interactions were observed, supporting its lower activity than **16b**.

### 2.4. In Vitro Mechanistic Evaluation: Topoisomerase II as a Target

**Figure 4:**
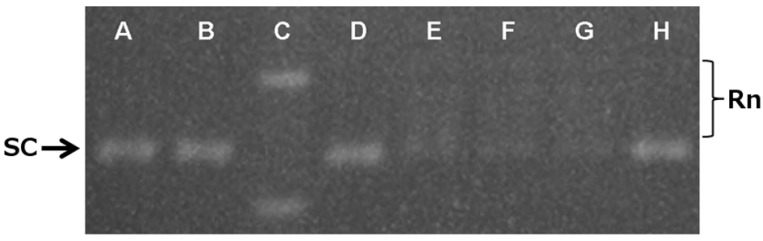
Effects of **16b** and **17b** on the inhibition of supercoiled DNA relaxation promoted by topoisomerase II. Supercoiled DNA (pRYG, 200 ng) was incubated with topoisomerase II (10 U) in the presence of the derivatives and then analyzed on an agarose gel without ethidium bromide. Lanes A and B, supercoiled DNA incubated with topoisomerase II and 100 µM of compounds **16b** or **17b**, respectively; lane C, ladder; lane D, supercoiled DNA without enzyme; lanes E,F,G, supercoiled DNA incubated with enzyme alone; lane H, supercoiled DNA with topoisomerase II in the presence of 100 μM VP-16. The arrow indicates the supercoiled (SC) DNA band, and the brackets indicate the topoisomer bands (Rn).

To confirm the theoretical data, we performed a topoisomerase II relaxation assay in the presence of **16b** and **17b** (Figure 4). The gel bands represent the different conformational forms of pRYG after the reaction catalyzed by topoisomerase II alone (lanes E, F and G) or pre-incubated with 100 µM **16 b**, **17b** or VP-16 (etoposide) (lanes A, B and H). The electrophoresis conditions of this experiment, specifically the absence of ethidium bromide, allowed the supercoiled (SC) form of pRYG to migrate as a single band on the gel. The addition of topoisomerase II unwinds the SC-producing topoisomers (Rn) and is shown in the gel as discrete bands that migrate slower than SC. In the presence of **16b**, **17b** or VP-16, only the SC band is observed, which indicates topoisomerase inhibition. These results support *in the* *in silico* docking evaluation pointing this enzyme a target for these new derivatives.

**Figure 5:**
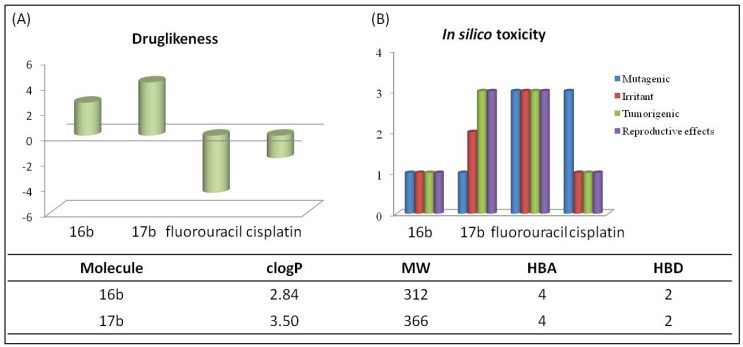
Comparison of **16b** and **17b** with anticancer marketed drugs, cisplatin and fluorouracil, (**A**) “Druglikeness”; and (**B**) *in silico* toxicity values calculated by using the Osiris Program and the physico-chemical parameters considering Lipinski’s rule-of-five paradigm.

### 2.5. In Silico Pharmacokinetic Analysis

In this work we also assessed **16b** and **17b** pharmacokinetic properties by using *in silico* evaluation. Because significant absorption is necessary for oral administration, we analyzed this derivative according to the “rule-of-five” developed by Lipinski and co-workers [25].The rule-of-five indicates the theoretical potential of a chemical compound to exhibit satisfactory oral bioavailability. The rule states that the most “druglike” molecules have a clog *P* ≤ 5, molecular weight (MW) ≤ 500, number of hydrogen bond acceptors (HBA) ≤ 10 and number of hydrogen bond donors (HBD) ≤ 5. Molecules violating more than one of these rules may show a low bioavailability profile. The results showed that compound **16b** and **17b** fulfilled the Lipinski “rule-of-five” (Figure 5). The “druglikeness test” calculation that evaluates the profile of the derivative as a drug, showed **16b** and **17b** with the better values than marketed drugs such as cisplatin and fluorouracil. Finally, according to our *in silico* toxicity evaluation of tumorigenic, irritant, mutagenic and reproductive effects, and compound **16b** showed low profile for these toxicity effects (Figure 5). It is important to note that the toxicity predicted herein neither is a fully reliable toxicity prediction nor guarantees that these compounds are completely free of any toxic effects. However, it reinforced the promising profile of 16b for further experimental evaluation.

## 3. Experimental

### 3.1. General Information

^1^H-NMR spectra were recorded on a Varian Unity Plus 300 spectrometer operating at 200.00 MHz, 300.00 MHz or 500.00 MHz (^1^H) and 50.0 MHz, 75.0 MHz or 125.0 MHz (^13^C), using CDCl_3_ or DMSO-*d*_6_ as the solvent. Chemical shifts were reported in parts per million (ppm) relative to the internal standard tetramethylsilane (TMS). Signals were designated as follows: brs, broad singlet; s, singlet; d, doublet; dd, doublet of doublets; t, triplet; q, quartet; m, multiplet. Hydrogen and carbon NMR spectra were typically obtained at room temperature. The two-dimensional experiments were acquired using standard Varian Associates automated programs for data acquisition and processing. The IR spectra were recorded on a Perkin-Elmer FT-IR 1600 spectrometer using potassium bromide pellets, and frequencies were expressed in cm^−1^. Mass spectra were obtained with ESI (MICRO-TOF BRUKER DALTONICS). The HPLC analysis was performed using a Dionex Ultimate 3000 HPLC System with a DAD Detector. Analyteevaluation was carried out with an Acclaim 120 C_18_column (3 μm, 150 × 4.6 mm), provided by Dionex (Sunnyvale, CA, USA).The melting points were determined with a Fisher-Johns apparatus and were uncorrected. All solvents and reagents were purchased from commercial sources: Sigma-Aldrich Brazil (São Paulo, Brazil), Acros Organics (Geel, Belgium) and Tedia Brazil (Rio de Janeiro, Brazil).

### 3.2. Synthesis

#### 3.2.1. General Procedure for the Synthesis of Anilinomethylenemalonates **8a**–**c**

A solution of the appropriate aniline (100 mmol), and diethyl ethoxymethylenemalonate(20.4 mL, 100 mmol) was heated under reflux for 3 h. The mixture was allowed to cool and then was poured into ice-cold water (100 g). The precipitate was collected by filtration and recrystallized from hexane to give derivatives **8a**–**c** [20,26,27].

*Diethyl 4-chloroanilinomethylenemalonate* (**8a**): yield: 22.4 g (90%) as white crystals; m.p.: 80–81 °C; ^1^H-NMR (200.00 MHz, CDCl_3_) δ (ppm): 11.01 (1H, d, *J* = 13.5 Hz, N-*H*), 8.46 (1H, d, *J* = 13.5 Hz, H-β), 7.37–7.31 (2H, m, H-2 and H-6), 7.11–7.04 (2H, m, H-3 and H-5), 4.32 (2H, q, *J* = 7.2 Hz, OC*H*_2_CH_3_), 4.26 (2H, q, *J* = 7.2 Hz, OC*H*_2_CH_3_), 1.39 (3H, t, *J* = 7.2 Hz, OCH_2_C*H*_3_), 1.34 (3H, t, *J* = 7.2 Hz, OCH_2_C*H*_3_); IR (KBr) ν (cm^−1^): 1721 and 1675 (C=O), 1262 (C-O).

*Diethyl 3-chloroanilinomethylenemalonate* (**8b**): yield: 22.6 g (91%) as white crystals; m.p.: 57–58 °C; ^1^H-NMR (200.00 MHz, CDCl_3_) δ (ppm): 10.99 (1H, d, *J* = 12.9 Hz, N-*H*), 8.45 (1H, d, *J* = 13.5 Hz, H-β), 7.10 (1H, dd, *J* = 2.1 and 0.9 Hz, H-2), 7.12–7.15 (1H, m, H-4), 7.30 (1H, t, *J* = 8.1 Hz, H-5), 7.01 (1H, ddd, *J* = 8.1, 2.1 and 1.2 Hz, H-6), 4.31 (2H, q, *J* = 7.2 Hz, OC*H*_2_CH_3_), 4.25 (2H, q, *J* = 7.2 Hz, OC*H*_2_CH_3_), 1.38 (3H, t, *J* = 7.2 Hz, OCH_2_C*H*_3_), 1.33 (3H, t, *J* = 7.2 Hz, OCH_2_C*H*_3_); IR (KBr) ν (cm^−1^): 1713 and 1688 (C=O), 1256 (C-O).

*Diethyl anilinomethylenemalonate* (**8c**): yield: 24.8 g (82%) as white crystals; m.p.: 46–48 °C; ^1^H-NMR (200.00 MHz, CDCl_3_) δ (ppm): 11.21 (1H, d, *J* = 13.2 Hz, N-*H*), 8.51 (1H, d, *J* = 13.5 Hz, H-β), 7.90–7.20 (5H, m, Ph), 4.32 (2H, q, *J* = 7.2 Hz, OC*H*_2_CH_3_), 4.25 (2H, q, *J* = 7.2 Hz, OC*H*_2_CH_3_), 1.38 (3H, t, *J* = 7.2 Hz, OCH_2_C*H*_3_), 1.33 (3H, t, *J* = 7.2 Hz, OCH_2_C*H*_3_); IR (KBr) ν (cm^−1^): 1718 and 1675 (C=O), 1261 (C-O).

#### 3.2.2. General Procedure for the Synthesis of Oxoquinolines **9a**–**c**

Anilinomethylenemalonates **8a**–**c** (3 g, 10.83 mmol) were refluxed for 30 min in diphenyl ether (30 mL), leading to crudeoxoquinolines **9a**–**c** which were recrystallized from dimethylformamide [20,21,26,27].

*3-Carbethoxy-6-chloro-1,4-dihydro-4-oxoquinoline* (**9a**): yield: 2.1 g (85%) as white crystals; m.p.: 295–296 °C; ^1^H-NMR (300.00 MHz, DMSO-*d*_6_) δ (ppm): 8.65 (1H, s, H-2), 8.22 (1H, d, *J* = 2.4 Hz, H-5), 7.85 (1H, dd, *J* = 9,0 and 2.4 Hz, H-7), 7.78 (1H, d, *J* = 8.7 Hz, H-8), 4.36 (2H, q, *J* = 6.9 Hz, OC*H*_2_CH_3_), 1.42 (3H, t, *J* = 6.9 Hz, OCH_2_C*H*_3_); IR (KBr) ν (cm^−1^): 3300–2800 (OH/NH), 1688 (C=O).

*3-Carbethoxy-7-chloro-1,4-dihydro-4-oxoquinoline* (**9b**): yield: 2.0 g (80%) as white crystals; m.p.: 293–294 °C; ^1^H-NMR (300.00 MHz, DMSO-*d*_6_) δ (ppm): 8.71 (1H, s, H-2), 8.26 (1H, d, *J* = 8.7 Hz, H-5), 7.59 (1H, dd, *J* = 9.0 and 2.1 Hz, H-6), 7.79 (1H, d, *J* = 2.1 Hz, H-8), 4.33 (2H, q, *J* = 6.9 Hz, OC*H*_2_CH_3_), 1.39 (3H, t, *J* = 6.9 Hz, OCH_2_C*H*_3_); IR (KBr) ν (cm^−1^): 3300–2800 (OH/NH), 1690 (C=O).

*3-Carbethoxy-1,4-dihydro-4-oxoquinoline* (**9c**): yield: 2.1 g (83%) as white crystals; m.p.: 268–269 °C; ^1^H-NMR (300.00 MHz, DMSO-*d*_6_) δ (ppm): 8.65 (1H, s, H-2), 8.22 (1H, d, *J* = 2.4 Hz, H-5), 7.85 (1H, dd, *J* = 9.0 and 2.4 Hz, H-7), 7.78 (1H, d, *J* = 8.7 Hz, H-8), 7.49 (1H, m, H_6_), 4.36 (2H, q, *J* = 6.9 Hz, OC*H*_2_CH_3_), 1.42 (3H, t, *J* = 6.9 Hz, OCH_2_C*H*_3_); IR (KBr) ν (cm^−1^): 3300–2800 (OH/NH), 1695 (C=O).

#### 3.2.3. General Procedure for the Synthesis of Oxoquinolines **10**–**18**

Oxoquinolines **9a**–**c**(8 mmol) were reacted with the appropriate amine (8 mmol) in diphenyl ether (30 mL) at 210 °C under magnetic stirring for 1 h. The resulting mixture was poured into petroleum ether. The obtained solid was filtered and recrystallized from dichloromethane/petroleum ether (1/1) to yield the derivatives listed below [28,29,30].

*4-Oxo-N'-(4-chlorobenzyl)-1,4-dihydroquinoline-3-carboxamide* (**10a**): yield: 1.4 g (96%) as a light brown solid; m.p.: 244–246 °C; ^1^H-NMR (500.00 MHz, DMSO-*d*_6_) δ (ppm): 10.43 (1H, t, *J* = 5.9 Hz, C=ON*H*), 8.75 (1H, s, H-2), 8.26 (1H, dd, *J* = 8.5 and 0.9 Hz, H-5), 7.77 (1H, ddd, *J* = 8.3, 6.8 and 1.5 Hz, H-7), 7.69 (1H, dd, *J* = 8.3 and 0.7 Hz, H-8), 7.48 (1H, ddd, *J* = 8.1, 6.8 and 1.3 Hz, H-6), 7.42-7.34 (4H, m, H-2', H-3', H-5' and H-6'), 4.56 (2H, d, *J* = 6.0 Hz, NHC*H*_2_); ^13^C-NMR (125.0 MHz, DMSO-*d*_6_) δ (ppm): 176.0, 164.5, 143.6, 139.1, 138.6, 132.5, 129.1, 128.2, 126.1, 125.3, 124.8, 118.9, 110.6, 41.3; IR (KBr) ν (cm^−1^): 3160 (N-H_amide_), 3066 (C-H_arom_), 1636 (C=O_ketone_), 1603 (C=O_amide_).

*6-Chloro-4-oxo-N'-(4-chlorobenzyl)-1,4-dihydroquinoline-3-carboxamide* (**10b**): yield: 1.2 g (85%) as a light brown solid; m.p.: 262–263 °C; ^1^H-NMR (500.00 MHz, DMSO-*d*_6_) δ (ppm): 10.58 (1H, t, *J* = 5.8 Hz, C=ON*H*), 8.79 (1H, s, H-2), 8.15 (1H, t, *J* = 1.5 Hz, H-7), 7.69 (1H, d, *J* = 1.8 Hz, H-5), 7.36 (5H, m, H-8, H-2', H-3', H-5' and H-6'), 4.53 (2H, d, *J* = 2.7 Hz, NHC*H*_2_); ^13^C-NMR (125.0 MHz, DMSO-*d*_6_) δ (ppm): 174.2, 165.2, 146.3, 140.6, 138.8, 131.6, 131.2, 129.6, 129.1, 128.7, 128.2, 128.1, 127.6, 110.3, 43.3; IR (KBr) ν (cm^−1^): 3150 (N-H_amide_), 3054 (C-H_arom_), 1631 (C=O_ketone_), 1605 (C=O_amide_).

*7-Chloro-4-oxo-N'-(4-chlorobenzyl)-1,4-dihydroquinoline-3-carboxamide* (**10c**): yield: 1.1 g (79%) as a light brown solid; m.p.: 220–221 °C; ^1^H-NMR (500.00 MHz, DMSO-*d*_6_) δ (ppm): 10.42 (1H, t, *J* = 5.8 Hz, C=ON*H*), 8.89 (1H, s, H-2), 8.34 (1H, d, *J* = 8.5 Hz, H-5), 7.85 (1H, m, H-8), 7.59 (1H, m, H-6), 7.48 (4H, d, *J* = 7.3 Hz, H-2', H-3', H-5' and H-6'), 4.71 (2H, d, *J* = 6.1 Hz, NHC*H*_2_); ^13^C-NMR (125.0 MHz, DMSO-*d*_6_) δ (ppm): 175.5, 164.3, 144.5, 139.9, 138.5, 137.2, 131.3, 129.1, 128.3, 127.6, 125.2, 124.7, 118.2, 111.2, 41.4; IR (KBr) ν (cm^−1^): 3150 (N-H_amide_), 3052 (C-H_arom_), 1655 (C=O_ketone_), 1627 (C=O_amide_).

*4-Oxo-N'-cyclohexyl-1,4-dihydroquinoline-3-carboxamide* (**11a**): yield: 0.4 g (30%) as a white solid; m.p.: 209–212 °C; ^1^H-NMR (500.00 MHz, DMSO-*d*_6_) δ (ppm): 10.10 (1H, d, *J* = 7.7 Hz, C=ON*H*), 8.75 (1H, s, H-2), 8.30 (1H, dd, *J* = 8.2 and 1.1 Hz, H-5), 7.81–7.77 (1H, m, H-7), 7.72 (1H, d, *J* = 8.1 Hz, H-8), 7.53-7.48 (1H, m, H-6), 3.89 (1H, m, H-1'), 1.89 (2H, m, H-2' _axial_ and H-6_axial_), 1.72 (2H, dd, *J* = 9.3 and 3.7 Hz, H-3' _axial_ and H-5'_axial_), 1.59 (1H, dd, *J* = 9.3 and 3.2 Hz, H-4'), 1.39 (5H, m, H-2'_equatorial_, H-3'_equatorial_, H-4'_equatorial_, H-5'_equatorial_, H-6'_equatorial_); ^13^C-NMR (125.0 MHz, DMSO-*d*_6_) δ (ppm): 176.1, 163.3, 143.3, 139.0, 132.4, 126.1, 125.3, 124.7, 118.8, 111.0, 46.6, 32.4, 25.2, 24.0; IR (KBr) ν (cm^−1^): 3433 (N-H_amide_), 1644 (C=O_ketone_), 1617 (C=O_amide_).

*6-Chloro-4-oxo-N'-cyclohexyl-1,4-dihydroquinoline-3-carboxamide* (**11b**): yield: 1.4 g (58%) as a white solid; m.p.: 140–142 °C; ^1^H-NMR (500.00 MHz, DMSO-*d*_6_) δ (ppm): 9.91 (1H, d, *J* = 7.8 Hz, C=ON*H*), 8.73 (1H, s, H-2), 8.17 (1H, d, *J* = 2.3 Hz, H-5), 7.77 (1H, dd, *J* = 8.8 and 2.3 Hz, H-7), 7.72 (1H, d, *J* = 8.8 Hz, H-8), 3.84 (1H, m, H-1'), 1.87 (2H, m, H-2'_axial_ and H-6'_axial_), 1.67 (2H, m, H-3'_axial_ and H-5'_axial_), 1.55 (2H, m, H-4'), 1.35 (4H, m, H-2'_equatorial_, H-3'_equatorial_, H-5'_equatorial_, H-6'_equatorial_); ^13^C-NMR (125.0 MHz, DMSO-*d*_6_) δ (ppm): 174.9, 162.9, 143.7, 137.7, 132.5, 129.4, 127.1, 124.2, 121.3, 111.3, 46.7, 32.4, 25.1, 24.0; IR (KBr) ν (cm^−1^): 3459 (N-H_amide_), 3063 (C-H_arom_), 1651 (C=O_ketone_), 1618 (C=O_amide_).

*7-Chloro-4-oxo-N'-cyclohexyl-1,4-dihydroquinoline-3-carboxamide* (**11c**): yield: 1.8 g (75%) as a white solid; m.p.: 140–141 °C; ^1^H-NMR (500.00 MHz, DMSO-*d*_6_) δ (ppm): 9.97 (1H, d, *J* = 7.7 Hz, C=ON*H*), 8.74 (1H, s, H-2), 8.22 (1H, d, *J* = 8.7 Hz, H-5), 7.72 (1H, s, H-8), 7.46 (1H, d, *J* = 8.7 Hz, H-6), 3.83 (1H, m, H-1'), 1.84 (2H, m, H-2'_axial_ and H-6'_axial_), 1.67 (2H, m, H-3'_axial_ and H-5'_axial_), 1.55 (1H, m, H-4'_axial_), 1.31 (5H, m, H-2'_equatorial_, H-3'_equatorial_, H-4'_equatorial_, H-5'_equatorial_, H-6'_equatorial_); ^13^C-NMR (125.0 MHz, DMSO-*d*_6_) δ (ppm): 175.5, 163.1, 144.5, 140.2, 137.0, 127.6, 125.0, 124.8, 118.4, 111.5, 46.7, 32.5, 25.2, 24.1; IR (KBr) ν (cm^−1^): 3460 (N-H_amide_), 3062 (C-H_arom_), 1630 (C=O_ketone_), 1570 (C=O_amide_).

*4-oxo-N'-(4-Chlorophenyl)-1,4-dihydroquinoline-3-carboxamide* (**12a**): yield: 1.3 g (96%) as a bright white solid; m.p.: >300 °C; ^1^H-NMR (500.00 MHz, DMSO-*d*_6_) δ (ppm): 12.54 (1H, s, C=ON*H*), 8.86 (1H, s, H-2), 8.32 (1H, dd, *J* = 8.3 and 1.0 Hz, H-5), 7.82–7.79 (1H, m, H-7), 7.76 (2H, d, *J* = 8.9 Hz, H-2' and H-6'), 7.74 (1H, d, *J* = 8.0 Hz, H-8), 7.53 (1H, m, H-6), 7.39 (2H, d, *J* = 8.5 Hz, H-3' and H-5'); ^13^C-NMR (125.0 MHz, DMSO-*d*_6_) δ (ppm): 176.2, 162.8, 144.1, 138.9, 137.5, 132.9, 128.7, 126.7, 125.7, 125.3, 125.2, 121.0, 119.1, 110.2; IR (KBr) ν (cm^−1^): 3209 (N-H _amide_), 3064 (C-H _arom_), 1665 (C=O _ketone_), 1626 (C=O _amide_).

*6-Chloro-4-oxo-N'-(4-chlorophenyl)-1,4-dihydroquinoline-3-carboxamide* (**12b**): yield: 1.3 g (98%) as a bright purple solid; m.p.: >300 °C; ^1^H-NMR (500.00 MHz, DMSO-*d*_6_) δ (ppm): 12.32 (1H, s, C=ON*H*), 8.86 (1H, s, H-2), 8.21 (1H, d, *J* = 2.3 Hz, H-5), 7.81 (1H, dd, *J* = 8.8 and 2.4 Hz, H-7), 7.76 (1H, d, *J* = 8.8 Hz, H-8), 7.73 (2H, d, *J* = 8.8 Hz, H-2' and H-3'), 7.39 (2H, d, *J* = 8.8 Hz, H-3' and H-5'); ^13^C-NMR (125.0 MHz, DMSO-*d*_6_) δ (ppm): 175.0, 162.5, 144.4, 137.6, 137.4, 132.9, 129.9, 128.7, 126.9, 126.8, 124.3, 121.5, 121.1, 110.6; IR (KBr) ν (cm^−1^): 3206 (N-H_amide_), 3063 (C-H_arom_), 1663 (C=O_ketone_), 1595 (C=O_amide_).

*4-oxo-N'-(4-Fluorophenyl)-1,4-dihydroquinoline-3-carboxamide* (**13a**): yield: 1.2 g (94%) as a bright purple solid; m.p.: >300 °C; ^1^H-NMR (500.00 MHz, DMSO-*d*_6_) δ (ppm): 12.47 (1H, s, C=ON*H*), 8.86 (1H, s, H-2), 8.32 (1H, dd, *J* = 8.3 and 1.4 Hz, H-5), 7.80 (1H, td, *J* = 8.3 and 1.4 Hz, H-7), 7.75 (2H, dd, *J* = 9.2 and 4.8 Hz, H-2' and H-6'), 7.73 (1H, d, *J* = 7.8 Hz, H-8), 7.53 (1H, td, *J* = 8.3 and 0.9 Hz, H-6) 7.18 (2H, t, *J* = 8.8 Hz, H-3' and H-5'); ^13^C-NMR (125.0 MHz, DMSO-*d*_6_) δ (ppm): 176.2, 162.7, 158.0 (d, ^1^*J*_C-F_ = 240.0 Hz), 144.0, 139.0, 135.1, 132.8, 125.8, 125.2 (d, ^3^*J*_C-F_ = 7.7 Hz), 121.2, 121.1, 119.1, 115.4 (d, ^2^*J*_C-F_ = 22.1 Hz), 110.3; IR (KBr) ν (cm^−1^): 3211 (N-H_amide_), 3067 (C-H_arom_), 1666 (C=O_ketone_), 1625 (C=O_amide_).

*7-Chloro-4-oxo-N'-(4-fluorphenyl)-1,4-dihydroquinoline-3-carboxamide* (**13b**): yield: 1.01 g (80%) as a bright gray solid; m.p.: >300 °C; ^1^H-NMR (500.00 MHz, DMSO-*d*_6_) δ (ppm): 12.28 (1H, s, C=ONH), 8.88 (1H, s, H-2), 8.28 (1H, d, *J* = 8.7 Hz, H-5), 7.77 (1H, d, *J* = 1.9 Hz, H-8), 7.73 (2H, dd, *J* = 9.1 and 4.9 Hz, H-2' and H-6'), 7.53 (1H, dd, *J* = 8.7 and 1.9 Hz, H-6), 7.18 (2H, d, *J* = 8.9 Hz, H-3' and H-5'); ^13^C-NMR (75.0 MHz, DMSO-*d*_6_) δ (ppm): 175.6, 162.3, 159.6, 156.4, 144.7, 139.8, 137.40, 134.9, 127.5, 125.4, 124.4, 121.3, 121.1, 118.3, 115.5, 115.2, 110.9; IR (KBr) ν (cm^−1^): 3069 (C-H_arom_), 1664 (C=O_ketone_), 1614 (C=O_amide_); HRMS-ESI (*m*/*z*): found for C_16_H_10_ClFN_2_O_2_ [M+H]^+^: 317.0488.

*4-oxo-N'-(4-Methoxyphenyl)-1,4-dihydroquinoline-3-carboxamide* (**14a**): yield: 1.2 g (86%) as a bright blue solid; m.p.: >300 °C; ^1^H-NMR (500.00 MHz, DMSO-*d*_6_) δ (ppm): 12.30 (1H, s, C=ON*H*), 8.85 (1H, s, H-2), 8.32 (1H, dd, *J* = 8.1 and 1.1 Hz, H-5), 7.79 (1H, td, *J* = 8.3 and 1.4 Hz, H-7), 7.73 (1H, d, *J* = 8.1 Hz, H-8), 7.64 (2H, d, *J* = 9.0 Hz, H-3' and H-5'), 7.52 (1H, t, *J* = 7.8 Hz, H-6), 6.93 (2H, d, *J* = 9.0 Hz, H-2' and H-6'), 3.75 (3H, s, OCH_3_); ^13^C-NMR (125.0 MHz, DMSO-*d*_6_) δ (ppm): 176.2, 162.3, 155.2, 143.8, 139.0, 132.8, 131.9, 125.8, 125.3, 125.0, 120.9, 119.0, 114.0, 110.6, 55.1; IR (KBr) ν (cm^−1^): 3212 (N-H _amide_), 3064 (C-H_arom_), 1659 (C=O_ketone_), 1606 (C=O_amide_).

*7-Chloro-4-oxo-N'-(4-methoxyphenyl)-1,4-dihydroquinoline-3-carboxamide* (**14b**): yield: 1.14 g (87%) as a bright purple solid; m.p.: 270–273 °C; ^1^H-NMR (500.00 MHz, DMSO-*d*_6_) δ (ppm): 12.12 (1H, s, C=ONH), 8.87 (1H, s, H-2), 8.29 (1H, d, *J* = 8.7 Hz, H-5), 7.78 (1H, d, *J* = 1.9 Hz, H-8), 7.63 (2H, d, *J* = 9.0 Hz, H-3' and H-5'), 7.53 (1H, dd, *J* = 8.7 and 1.9 Hz, H-6), 6.93 (2H, d, *J* = 9.0 Hz, H-2' and H-6'), 3.87 (3H, s, OCH_3_); ^13^C-NMR (75.0 MHz, DMSO-*d*_6_) δ (ppm): 175.6, 161.9, 155.2, 146.5, 139.8, 137.3, 131.7, 127.5, 125.3, 124.4, 120.9, 118.2, 114.0, 111.2, 55.0; IR (KBr) ν (cm^−1^): 3201 (N-H_amide_), 3070 (C-H_arom_), 1657 (C=O_ketone_), 1621 (C=O_amide_); HRMS-ESI (*m*/*z*): found for C_17_H_13_ClN_2_O_3_ [M+H]^+^: 329.0687.

*4-oxo-N'-Phenyl-1,4-dihydroquinoline-3-carboxamide* (**15a**): yield: 0.9 g (79%) as a light brown solid; m.p.: >300 °C; ^1^H-NMR (500.00 MHz, DMSO-*d*_6_) δ (ppm): 12.48 (1H, s, C=ON*H*), 8.90 (1H, s, H-2), 8.38 (1H, dd, *J* = 8.1 and 0.9 Hz, H-5), 7.87-782 (1H, m, H-7), 7.78 (1H, m, H-8, H-2' and H-6'), 7.57 (1H, m, H-6), 7.40 (2H, t, *J* = 7.9 Hz, H-3' and H-5'), 7.13 (1H, t, *J* = 7.4 Hz, H-4'); ^13^C-NMR (125.0 MHz, DMSO-*d*_6_) δ (ppm): 176.2, 162.7, 143.9, 139.0, 138.7, 132.8, 128.8, 125.9, 125.4, 125.1, 123.3, 119.5, 119.0, 110.6; IR (KBr) ν (cm^−1^): 3255 (N-H_amide_), 3065 (C-H_arom_), 1667 (C=O_ketone_), 1618 (C=O_amide_).

*6-Chloro-4-oxo-N'-phenyl-1,4-dihydroquinoline-3-carboxamide* (**15b**): yield: 1.0 g (85%) as a bright white solid; m.p.: >300 °C; ^1^H-NMR (500.00 MHz, DMSO-*d*_6_) δ (ppm): 12.27 (1H, s, C=ON*H*), 8.88 (1H, s, H-2), 8.23 (1H, d, *J* = 2.4 Hz, H-5), 7.83 (1H, dd, *J* = 8.8 and 2.4 Hz, H-7), 7.77 (1H, d, *J* = 8.8 Hz, H-8), 7.71 (2H, d, *J* = 8.4 Hz, H-2' and H-6'), 7.36 (2H, t, *J* = 7.9 Hz, H-3' and H-5'), 7.12-7.07 (1H, m, H-4'); ^13^C-NMR (125.0 MHz, DMSO-*d*_6_) δ (ppm): 175.1, 162.4, 144.5, 138.6, 137.8, 132.9, 128.9, 128.9, 127.0, 124.4, 123.4, 121.6, 119.6, 110.9; IR (KBr) ν (cm^−1^): 3204 (N-H_amide_), 3058 (C-H_arom_), 1653 (C=O_ketone_), 1597 (C=O_amide_).

*7-Chloro-4-oxo-N'-phenyl-1,4-dihydroquinoline-3-carboxamide* (**15c**): yield: 1.0 g (83%) as a light brown solid; m.p.: >300 °C; ^1^H-NMR (500.00 MHz, DMSO-*d*_6_) δ (ppm): 12.28 (1H, s, C=ON*H*), 8.89 (1H, s, H-2), 8.29 (1H, d, *J* = 8.7 Hz, H-5), 7.77 (1H, d, *J* = 1.8 Hz, H-8), 7.71 (1H, d, *J* = 7.6 Hz, H-2' and H-6'), 7.53 (1H, dd, *J* = 8.7 and 1.9 Hz, H-6), 7.36 (2H, t, *J* = 7.9 Hz, H-3' and H-5'), 7.09 (1H, t, *J* = 7.4 Hz, H-4'); ^13^C-NMR (125.0 MHz, DMSO-*d*_6_) δ (ppm): 175.7, 162.4, 144.8, 139.8, 138.6, 137.5, 128.9, 127.7, 125.5, 124.6, 123.4, 119.6, 118.3, 111.2; IR (KBr) ν (cm^−1^): 3204 (N-H_amide_), 3069 (C-H_arom_), 1662 (C=O_ketone_), 1618 (C=O_amide_).

*4-Oxo-N'-(p-tolyl)-1,4-dihydroquinoline-3-carboxamide* (**16a**): yield: 1.2 g (94%) as a bright rose solid; m.p.: >300 °C; ^1^H-NMR (500.00 MHz, DMSO-*d*_6_) δ (ppm): 12.36 (1H, s, C=ON*H*), 8.84 (1H, s, H-2), 8.32 (1H, d, *J* = 8.2 Hz, H-5), 7.80 (1H, t, *J* = 8.3 Hz, H-7), 7.73 (1H, d, *J* = 8.2 Hz, H-8), 7.60 (2H, d, *J* = 8.3 Hz, H-3' and H-5'), 7.52 (1H, t, *J* = 7.9 Hz, H-6), 7.16 (2H, d, *J* = 8.3 Hz, H-2' and H-6'), 2.27 (3H, s, C*H*_3_); ^13^C-NMR (125.0 MHz, DMSO-*d*_6_) δ (ppm): 176.2, 162.5, 143.9, 139.0, 136.2, 132.8, 132.7, 129.3, 125.8, 125.3, 125.1, 119.5, 119.1, 110.6, 20.3; IR (KBr) ν (cm^−1^): 3209 (N-H_amide_), 3070 (C-H_arom_), 1663 (C=O_ketone_), 1604 (C=O_amide_).

*6-Chloro-4-oxo-N'-(p-tolyl)-1,4-dihydroquinoline-3-carboxamide* (**16b**): yield: 1.19 g (96%) as a bright gray solid; m.p.: >300 °C; ^1^H-NMR (500.00 MHz, DMSO-*d*_6_) δ (ppm): 12.18 (1H, s, C=ONH), 8.87 (1H, s, H-2), 8.22 (1H, dd, *J* = 2.3 and 0.7 Hz, H-5), 7.82 (1H, dd, *J* = 8.9 and 2.3 Hz, H-7), 7.76 (1H, dd, *J* = 8.9 and 0.7 Hz, H-8), 7.58 (2H, d, *J* = 8.6 Hz, H-3' and H-5'), 7.16 (2H, d, *J* = 8.6 Hz, H-2' and H-6'), 2.27 (3H, s, CH_3_); ^13^C-NMR (75.0 MHz, DMSO-*d*_6_) δ (ppm): 174.9, 162.1, 144.1, 137.7, 136.0, 132.8, 132.3, 129.7, 129.2, 126.9, 124.2, 121.5, 119.4, 110.9, 20.3; IR (KBr) ν (cm^−1^): 3155 (N-H_amide_), 3065 (C-H_arom_), 1662 (C=O_ketone_), 1605 (C=O_amide_); HRMS-ESI (*m*/*z*): found for C_17_H_13_ClN_2_O_2_ [M + H]^+^: 313.0738; HPLC chromatogram (see Supporting Information).

*7-Chloro-4-oxo-N'-(p-tolyl)-1,4-dihydroquinoline-3-carboxamide* (**16c**): yield: 1.07 g (86%) as a green solid; m.p.: >300 °C; ^1^H-NMR (500.00 MHz, DMSO-*d*_6_) δ (ppm): 12.18 (1H, s, C=ONH), 8.87 (1H, s, H-2), 8.28 (1H, d, *J* = 8.8 Hz, H-5), 7.76 (1H, d, *J* = 1.8 Hz, H-8), 7.59 (2H, d, *J* = 8.2 Hz, H-3' and H-5'), 7.52 (1H, dd, *J* = 8.8 and 1.8 Hz, H-6), 7.15 (2H, d, *J* = 8.2 Hz, H-2' and H-6'), 2.27 (3H, s, CH_3_); ^13^C-NMR (75.0 MHz, DMSO-*d*_6_) δ (ppm): 175.5, 162.0, 139.7, 137.3, 135.9, 132.2, 129.1, 127.5, 125.3, 124.4, 119.4, 118.1, 111.1, 20.2; IR (KBr) ν (cm^−1^): 3205 (N-H_amide_), 3069 (C-H_arom_), 1681 (C=O_ketone_), 1602 (C=O_amide_); HRMS-ESI (*m*/*z*): found for C_17_H_13_ClN_2_O_2_ [M+H]^+^: 313.0738.

*4-Oxo-N'-(2,5-dichlorophenyl)-1,4-dihydroquinoline-3-carboxamide* (**17a**): yield: 1.01 g (66%) as a light brown solid; m.p.: >300 °C; ^1^H-NMR (500.00 MHz, DMSO-*d*_6_) δ (ppm): 12.98 (1H, s, C=ONH), 8.86 (1H, s, H-2), 8.70 (1H, d, *J* = 2.6 Hz, H-6'), 8.32 (1H, dd, *J* = 8.2 and 1.0 Hz, H-5), 7.80 (1H, td, *J* = 8.3 and 1.3 Hz, H-7), 7.72 (1H, d, *J* = 8.2 Hz, H-8), 7.56-7.50 (2H, m, H-3' and H-6), 7.15 (1H, dd, *J* = 8.6 and 2.6 Hz, H-4'); ^13^C-NMR (75.0 MHz, DMSO-*d*_6_) δ (ppm): 176.1, 163.5, 144.5, 138.9, 136.99, 133.0, 131.7, 130.4, 125.8, 125.4, 125.3, 123.6, 120.7, 120.6, 119.1, 109.8; IR (KBr) ν (cm^−1^): 3264 (N-H_amide_), 3023 (C-H_arom_), 1677 (C=O_ketone_), 1634 (C=O_amide_); HRMS-ESI (*m*/*z*): found for C_16_H_10_Cl_2_N_2_O_2_ [M+H]^+^: 333.0192.

*6-Chloro-4-oxo-N'-(2,5-dichlorophenyl)-1,4-dihydroquinoline-3-carboxamide* (**17b**): yield: 1.43 g (98%) as a bright beige solid; m.p.: >300 °C; ^1^H-NMR (500.00 MHz, DMSO-*d*_6_) δ (ppm): 12.74 (1H, s, C=ONH), 8.85 (1H, s, H-2), 8.65 (1H, d, *J* = 2.9 Hz, H-6'), 8.20 (1H, d, *J* = 1.9 Hz, H-5), 7.79 (1H, dd, *J* = 8.8 and 1.9 Hz, H-7), 7.73 (1H, d, *J* = 8.8 Hz, H-8), 7.52 (1H, d, *J* = 8.8 Hz, H-3'), 7.14 (1H, dd, *J* = 8.8 and 2.9 Hz, H-4'); ^13^C-NMR (75.0 MHz, DMSO-*d*_6_) δ (ppm): 175.0, 163.1, 144.8, 137.6, 136.8, 133.0, 131.8, 130.4, 130.0, 126.8, 124.4, 123.7, 121.5, 120.6, 110.2; IR (KBr) ν (cm^−1^): 3213 (N-H_amide_), 3089 (C-H_arom_), 1659 (C=O_ketone_), 1619 (C=O_amide_); HRMS-ESI (*m*/*z*): found for C_16_H_9_Cl_3_N_2_O_2_ [M+H]^+^: 366.8970; HPLC chromatogram (Supporting Information).

*7-Chloro-4-oxo-N'-(2,5-dichlorophenyl)-1,4-dihydroquinoline-3-carboxamide* (**17c**): yield: 0.92 g (63%) as a bright beige solid; m.p.: >300 °C; ^1^H-NMR (500.00 MHz, DMSO-*d*_6_) δ (ppm): 12.33 (1H, s, C=ONH), 8.86 (1H, s, H-2), 8.67 (1H, d, *J* = 2.6 Hz, H-6'), 8.28 (1H, d, *J* = 9.2 Hz, H-3'), 7.75 (1H, d, *J* = 1.9 Hz, H-8), 7.54 (1H, d, *J* = 8.6 Hz, H-5), 7.50 (1H, dd, *J* = 8.6 and 1.9 Hz, H-6), 7.15 (1H, dd, *J* = 8.6 and 2.6 Hz, H-4'); ^13^C-NMR (75.0 MHz, DMSO-*d*_6_) δ (ppm):175.5, 163.0, 145.2, 139.7, 137.4, 136.8, 131.7, 130.3, 127.5, 125.4, 124.4, 123.5, 120.7, 120.6, 118.3, 110.4; IR (KBr) ν (cm^−1^): 3204 (N-H_amide_), 3065 (C-H_arom_), 1680 (C=O_ketone_), 1652 (C=O_amide_); HRMS-ESI (*m*/*z*): found for C_16_H_9_Cl_3_N_2_O_2_ [M+H]^+^: 366.9850.

*4-Oxo-N'-(2,5-dimethoxyphenyl)-1,4-dihydroquinoline-3-carboxamide* (**18a**): yield: 0.85 g (57%) as a gray solid; m.p.: 250–251 °C; ^1^H-NMR (500.00 MHz, DMSO-*d*_6_) δ (ppm): 12.50 (1H, s, C=ONH), 8.89 (1H, s, H-2), 8.32 (1H, dd, *J* = 8.0 and 1.0 Hz, H-5), 8.21 (1H, d, *J* = 3.3 Hz, H-6'), 7.80 (1H, td, *J* = 8.3 and 1.3 Hz, H-7), 7.71 (1H, d, *J* = 7.6 Hz, H-8), 7.50 (1H, td, *J* = 8.3 and 1.3 Hz, H-6), 6.97 (1H, d, *J* = 8.9 Hz, H-3'), 6.59 (1H, dd, *J* = 8.6 and 3.0 Hz, H-4'), 3.87 (3H, s, OCH_3_), 3.71 (3H, s, OCH_3_); ^13^C-NMR (75.0 MHz, DMSO-*d*_6_) δ (ppm): 175.9, 162.8, 153.0, 143.9, 142.7, 138.9, 132.7, 129.1, 126.0, 125.4, 125.0, 118.9, 111.6, 110.8, 107.0, 106.8; IR (KBr) ν (cm^−1^): 3213 (N-H_amide_), 3058 (C-H_arom_), 1666 (C=O_ketone_), 1633 (C=O_amide_); HRMS-ESI (*m*/*z*): found for C_18_H_16_N_2_O_4_ [M+H]^+^: 325.1183.

*6-Chloro-4-oxo-N'-(2,5-dimethoxyphenyl)-1,4-dihydroquinoline-3-carboxamide* (**18b**): yield: 0.62 g (44%) as a bright gray solid; m.p.: 256–258 °C; ^1^H-NMR (500.00 MHz, DMSO-*d*_6_) δ (ppm): 12.41 (1H, s, C=ONH), 8.87 (1H, s, H-2), 8.21 (1H, d, *J* = 2.9 Hz, H-6'), 8.26 (1H, d, *J* = 1.9 Hz, H-5), 7.81 (1H, dd, *J* = 8.9 and 2.3 Hz, H-7), 7.75 (1H, d, *J* = 8.9 Hz, H-8), 6.98 (1H, d, *J* = 8.9 Hz, H-3'), 6.60 (1H, dd, *J* = 8.9 and 2.9 Hz, H-4'), 3.87 (3H, s, OCH_3_), 3.71 (3H, s, OCH_3_); ^13^C-NMR (75.0 MHz, DMSO-*d*_6_) δ (ppm): 174.7, 162.6, 153.0, 144.9, 142.6, 138.2, 132.6, 129.6, 129.0, 127.1, 124.4, 121.8, 111.5, 111.0, 107.0, 106.8, 56.4, 55.2; IR (KBr) ν (cm^−1^): 3154 (N-H_amide_), 3090 (C-H_arom_), 1721 (C=O_ketone_), 1695 (C=O_amide_); HRMS-ESI (*m*/*z*): found for C_18_H_15_ClN_2_O_4_ [M+H]^+^: 359.0793.

*7-Chloro-4-oxo-N'-(2,5-dimethoxyphenyl)-1,4-dihydroquinoline-3-carboxamide* (**18c**): yield: 0.64 g (45%) as a gray solid; m.p.: 261–262 °C; ^1^H-NMR (500.00 MHz, DMSO-*d*_6_) δ (ppm): 12.38 (1H, s, C=ONH), 8.87 (1H, s, H-2), 8.31 (1H, d, *J* = 8.6 Hz, H-5), 8.19 (1H, d, *J* = 3.3 Hz, H-6'), 7.75 (1H, d, *J* = 1.9 Hz, H-8), 7.51 (1H, dd, *J* = 8.6 and 1.9 Hz, H-6), 6.98 (1H, d, *J* = 8.9 Hz, H-3'), 6.59 (1H, dd, *J* = 8.9 and 2.9 Hz, H-4'), 3.87 (3H, s, OCH_3_), 3.71 (3H, s, OCH_3_); ^13^C-NMR (75.0 MHz, DMSO-*d*_6_) δ (ppm): 175.4, 162.5, 153.0, 144.9, 142.7, 139.9, 137.2, 128.9, 127.7, 125.3, 124.7, 118.3, 111.6, 111.4, 107.0, 106.8, 56.4, 55.2; IR (KBr) ν (cm^−1^): 3401 (N-H_amide_), 3071 (C-H_arom_), 1654 (C=O_ketone_), 1623 (C=O_amide_); HRMS-ESI (*m*/*z*): found for C_18_H_15_ClN_2_O_4_ [M+H]^+^: 359.0793.

### 3.3. Instrumental Parameters for HPLC

The HPLC analysis of the active 4-oxoquinoline-3-carboxamide derivatives **16b** and **17b** was carried out by injecting 20 μL of a 25.6 μmol·L^−1^ standard solution of **16b** and 21.7 μmol·L^−1^ standard solution of **17b**, using acetonitrile as mobile phase pumped at a flow rate of 1 mL·min^−1^. The temperature of the column was set at 20 °C (see Supporting Information).

### 3.4. Molecular Docking Studies

Docking studies were performed using the GOLD program on a Windows-based PC. The three-dimensional structure of **16b** and **17b** were built and minimized to the PM6 level on the molecular modeling program Spartan’10 (Wavefunction Inc., Irvine, CA, USA). The coordinates of the protein crystal structure were obtained from the Protein Data Bank (PDB code 3QX3). The ligand etoposide and solvent molecules were removed. Hydrogen atoms were added, and non-hydrogen atoms were merged with the polar respective carbon atoms. Taking the prepared protein and ligand, GOLD docking calculations were performed using standard parameters. The scoring function used during GOLD docking was Goldscore. For each of the 100 independent genetic algorithm (GA) runs, with a selection pressure of 1.1, 10.000 GA operations were performed on a set of five islands with a population size of 100 individuals. Default operator weights were used for crossover, mutation, and migration of 95, 95, and 10, respectively. To expedite the calculations, the GA docking was terminated when the top three solutions were within 1.5 Å RMSD. All other values were set to the default [31,32]. Finally, eighty top ranked positions (or conformations) were saved.

### 3.5. DNA Relaxation Assay

Human topoisomerase IIα (TopoIIα) was obtained from TopoGEN, Inc. (Columbus, OH, USA). Proteinase K from *Tritirachium album* was obtained from Sigma-Aldrich, Inc. (St. Louis, MO, USA) and was dissolved in DNase free water. A TAE gel electrophoresis buffer was used in this assay.

The inhibitory effects of the derivatives on human TopoIIα were measured using a Eukaryotic Topoisomerase II Drug Screening Kit (TopoGEN, Inc.). All derivatives were dissolved in DMSO immediately prior to testing.

The substances were tested at a fixed concentration of 0.1 mM. This assay concentration was chosen based on the effective concentration of the VP-16 (etoposide) standard (0.1 mM), as recommended by the kit manufacturer (TopoGen). To ensure that DMSO did not interfere in the experiment, different concentrations of DMSO were tested. There was no interference in enzyme function with concentrations from 0%–5% DMSO. Supercoiled plasmid DNA (pRYG, 200 ng) was incubated with human TopoIIα (10 U) at 37 °C for 30 min in relaxation buffers in the absence or presence of derivatives (final volume is 20 μL). The order of reagent addition was buffer, test derivative, Topo IIα and finally DNA.

The assay samples were analyzed by electrophoresis on a 1% agarose gel without EtdBr (25 V, 18 h, room temperature) in TAE buffer, followed by staining in 0.5 μg/mL of EtdBr to allow for observation of the DNA bands under UV light.

### 3.6. In Silico Pharmacokinetics and Toxicity Analysis

For this analysis, we used Osiris Property Explorer [33] to predict the physicochemical properties (cLogP, solubility), toxicity and “druglikeness” of the most actives derivatives, **16b** and **17b**. Values of “druglikeness” are based on the frequency of occurrence of each fragment of the molecule in commercial drugs, and the drug score evaluates the derivative’s potential to qualify as a drug and is related to topological descriptors, fingerprints of molecular “druglikeness”, structural keys and other properties such as cLog P, log S and molecular weight.

### 3.7. Anticancer Assays

#### 3.7.1. Cytotoxicity against Cancer Cell Lines

The 4-oxoquinoline-3-carboxamide derivatives (0.3125–20 μM) were tested for cytotoxic activity against three cancer cell lines: HCT-116 (colon), ACP03 (gastric), and MDAMB-231 (breast) (Table 1). Active derivatives were also tested against a normal fibroblast cell line (MRC-5). All cell lines were maintained in DMEM medium supplemented with 10% fetal bovine serum, 2 mM glutamine, 100 U/mL penicillin, and 100 µg/mL streptomycin and were incubated at 37 °C with 5% CO_2_. Each derivative was dissolved with DMSO to a concentration of 10 mM. The final concentration of DMSO in the culture medium was kept at a constant value below 0.1% (*v*/*v*). The derivatives (0.3125–20 µM) were incubated with the cells for 72 h. The negative control contained the same amount of DMSO (0.001% maximum). Cell viability was determined by the reduction of the yellow dye 3-(4,5-dimethyl-2-thiazol)-2,5-diphenyl-2*H*-tetrazolium bromide (MTT) to a blue formazan product as described by Mosmann [34].

#### 3.7.2. Cell Membrane Disruption

The assay was performed in 96-well plates using a 2% mouse erythrocyte suspension in 0.85% NaCl containing 10 mM CaCl_2_. Derivatives **10**–**18**, diluted as described above, were tested at 250 µg/mL. After incubation at room temperature for 1 h and centrifugation, the supernatant was removed, and the liberated hemoglobin was measured spectrophotometrically at 540 nm. DMSO was used as a negative control, and Triton X-100 (1%) was used as a positive control.

#### 3.7.3. Analysis of the Results

In the MTT experiments, the results were analyzed according to their means and standard errors in the program GraphPad Prism. Each sample was analyzed in triplicate.

## 4. Conclusions

In this work, we synthesized a new series of 4-oxoquinoline derivatives that exhibited significant antitumor activity against gastric cancer cells. *In vitro* studies showed that the mechanism of action of **16b** is related to topoisomerase II inhibition, which was corroborated by *in silico* studies. **16b** and **17b** were more selective to cancer cells compared to doxorubicin, and these results may contribute to the design of novel anticancer derivatives with fewer side effects.

## Supplementary Materials

Supplementary materials can be accessed at: http://www.mdpi.com/1420-3049/19/5/6651/s1.
