# Supplementary material for: Synthesis, Cytotoxicity and Mechanistic Evaluation of 4-Oxoquinoline-3-carboxamide Derivatives: Finding New Potential Anticancer Drugs
Source: Molecules. 2014 May 22;19(5):6651–70. doi: 10.3390/molecules19056651 (PMC6271384; doi:10.3390/molecules19056651)
Supplement: Supplementary file 1 [file molecules-19-06651-s001.pdf]

Supporting Information

Chromatograms

Figure S1. (**A**) HPLC Chromatogram of **16b**; (**B**) HPLC Chromatogram of **17b**.

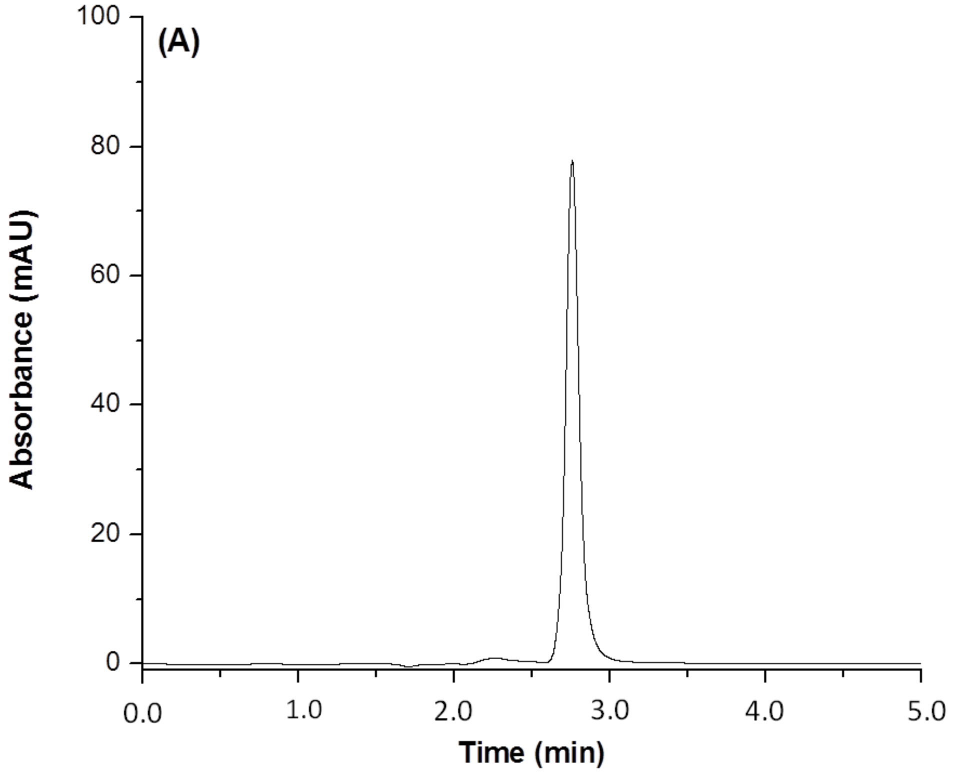

No.	*Peakname*	*Ret. Time min*	*Area mAU*min*	*Height mAU*	*Match*	*RSD Match %*	*PPI nm*	*RSD PPI %*
1	n.a.	2,757	8,1680	77,543	100	0,57	244,1	0,17
Total:			8,1680	77,5433				

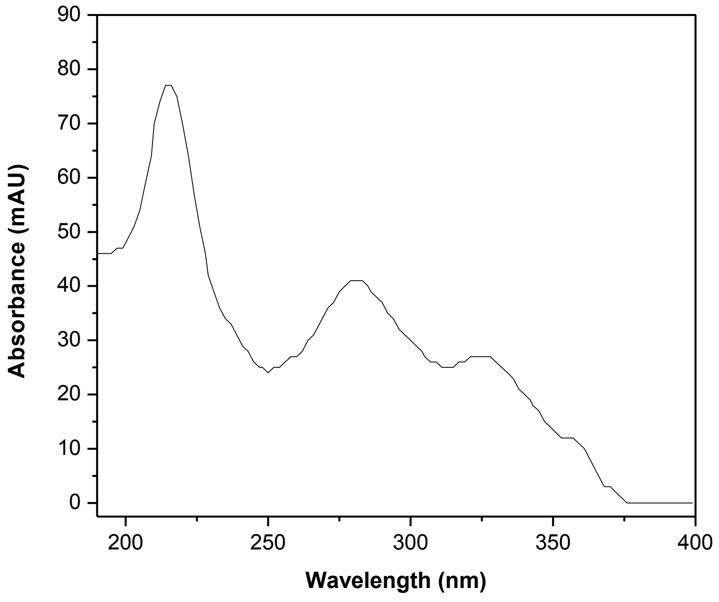

Absorption spectra observed in the maximum of the chromatographic peak.

Figure S1. *Cont.*

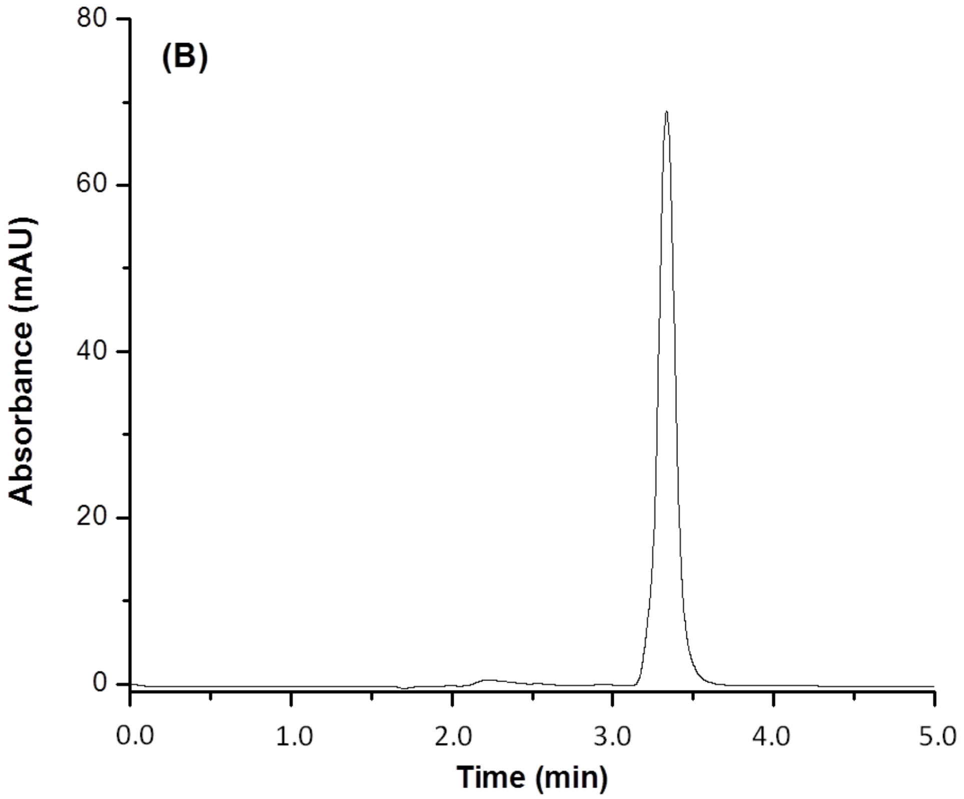

No.	*Peakname*	*Ret. Time min*	*Area mAU*min*	*Height mAU*	*Match*	*RSD Match %*	*PPI nm*	*RSD PPI %*
1	n.a.	3,333	8,8987	68,934	100	0,18	244,6	0,18
Total:			8,8987	68,9343				

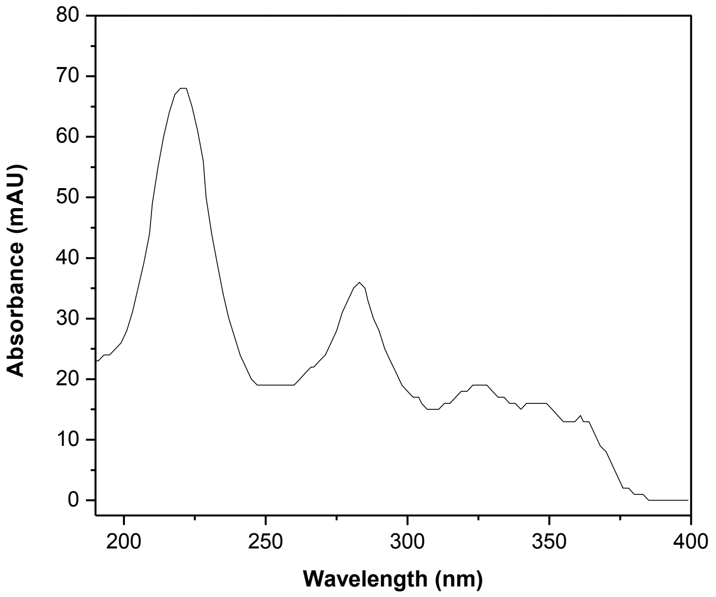

Absorption spectra observed in the maximum of the chromatographic peak.
